# The Effectiveness of Home Water Purification Systems on the Amount of Fluoride in Drinking Water

**Published:** 2015-09

**Authors:** Behrooz Eftekhar, Masoume Skini, Milad Shamohammadi, Jaber Ghaffaripour, Firoozeh Nilchian

**Affiliations:** a Dept. of Endodontic, School of Dentistry, Ahwaz Jondishapoor University of Medical Sciences, Ahwaz, Iran.; b Postgraduate Student, Dept. of Endodontic, School of Dentistry, Ahwaz Jondishapoor University of Medical Sciences, Ahwaz, Iran.; c DDS, School of Dentistry, Ahwaz Jondishapoor University of Medical Sciences, Ahwaz, Iran.; d Dental Students Research Center, Dept. of Dental Public Health, School of Dentistry, Isfahan University of Medical Sciences, Isfahan, Iran.

**Keywords:** Water Purification, Fluoride, Spectrophotometry

## Abstract

**Statement of the Problem:**

Water purification systems for domestic use have drawn significant attention over the past few years. This can be related to the improvement of public health and concern for water contamination.

**Purpose:**

The aim of this study was to evaluate whether home water purification systems eliminate the essential materials such as fluoride besides filtrating the heavy ions and other unwanted particles out of water.

**Materials and Method:**

In this experimental study, six most frequently used commercial brands of water purifiers were evaluated and compared. Specimens were collected right before and after setting up the device, and 6 months later. Then, spectrophotometry (the Harrison device) was performed to compare fluoride clearance by each home water cleaner device.

**Results:**

Based on the data collected from all water purification devices in different locations, the amount of fluoride was significantly different before and right after using home water purifier and six months later (*p*= 0.001 and *p*= 0.00, respectively).

**Conclusion:**

The filtration of water significantly decreased its fluoride concentration. The fluoride content of purified water was approximately as much as zero in some cases.

## Introduction

Fluoride is a natural element branched from Fluorine. This element can be found in all sorts of water and soil. Out of every kilogram of outer layer of earth, 0.3 gram is fluoride. Mineral waters have more amount of this element compared to other sources.(1)

About 60 years ago, Grand Rapids in Michigan State was the first city in which fluoride supplement was synthetically added to tap water. In US, adding fluoride to community water supplies of many cities has improved the oral health of millions of American citizens.(2)

Fluoridation of community water supplies is adding a specific amount of fluoride (0.7-1.2 ppm) to water in order to reduce the risk of dental caries. By 2002, almost 170 million Americans were provided with this privilege.(3)

Since most of the systemic fluoride is provided through tap water to population, many policies have been established to add fluoride to community water regarding its benefits for teeth and bones.(4)

In regions and countries that do not have water-fluoridation technology, there are natural supplements as previously mentioned. For example, Iran has many mineral water supplies that contain considerable amounts of fluoride. Amount of fluoride in natural mineral waters depends on weather conditions; the warmer the weather is, the higher the amount of fluoride can be detected. Mineral waters in southern regions that have warmer weather contain more fluoride. In Iran, the highest amount of fluoride has been found in southeast and northeast areas. 

Water purification systems for domestic use have drawn much of attention over the past few years. This can be related to improvement of public health and concerns for water contamination. There are several types of home water purification systems that can be categorized into 3 different groups(5) as filtered systems, systems using UV irradiation, and ion-exchange systems.

The aim of this study was to find out whether domestic water purification systems could eliminate the essential materials such as fluoride besides filtrating the heavy ions and other unwanted particles out of water. 

## Materials and Method

In this study, 6 frequently used commercial brands of water purifiers in Ahwaz were compared. The commercial brands evaluated in the current study were CCK (Ceramic and Ceramic/Carbon Cartridges ; RTX-TS DLM filters, Korea), Soft Water (Ceramic Candles; Alpine TJ Series filters, W9332420, USA), Alkusar (Special media cartridges filters; PRB50-IN, USA), Puricom (Special media cartridges filters; Watts 4.5" x 10" Dual Housing, Korea), Water Safe (Granular Carbon Cartridges filters; LCV (Lead, Cysts, VOC's) (Carbon Block Filter Cartridges, Australia), and Aquafresh (Sediment String-Wound; Poly Spun and Pleated Washable Cartridges filters, K5520, USA). The main drinking water supply for Ahwaz is provided by governmental companies. After making arrangement with certain companies that supported these brands, the devices were setup in 6 different regions of Ahwaz. Samples were collected before and right after setting up the device. To reduce the errors and elevate the accuracy of the module, 5 samples were taken from each device. Another sample was collected from each single device 6 months later. A total of 64 samples were collected including 32 unfiltered (control) and 32 filtered samples of tap water (experimental) from 6 regions in Ahwaz. Fluoride sampling kits (Spands; EW-99574-08Hach^®^ Test Kits, USA) were used to test the amount of fluoride in sample waters. Samples were all collected in polyethylene sampling containers and were then coded. Spectrophotometry (AvaSpec-ULS2048L- USB2 UARS spectrometer, USA) was performed. In order to measure the characteristics of individual molecules, a mass spectrometer converted them to ions so that they could be moved about and manipulated by external electric and magnetic fields.

Atmospheric pressure was around 760 torr (mm of mercury). The pressure under which ions may be handled is roughly 10^-5^ to 10^-8^ torr (less than a billionth of an atmosphere). By varying the strength of the magnetic field, ions of different mass can be focused progressively on a detector fixed at the end of a curved tube and also under a high vacuum.

Latin alphabetic words were used to code each commercial device. Numbers were used for samples obtained before and after setting the device.(6)

The results were analyzed by using paired sample t-test, with alpha (ɑ) set at 0.05. 

## Results

The amount of fluoride in water before and after using six brands of water purifier device is summarized in Table 1.

**Table 1:** The amount of fluoride before and after installing water purifier devices

** Fluoride Amount** **Purifier Device**	**Before Installing Water Purifier (ppm)**	**After Installing Water Purifier (ppm)**
Alkusar	0.283	0.035
Aquafresh	0.310	0.20
Soft Water	0.315	0.010
Water Safe	0.285	0.025
Puricom	0.312	0.018
CCK	0.385	0.010

Based on the data gathered from all water purification devices set in different regions, the level of fluoride was significantly different before and after using home water purifier (*p*= 0.001). It was found that home water purifiers nearly eliminated fluoride from tap water. Table 2 represents the results of t-test.

**Table 2:** Comparison of different study groups with t-test

	**Mean**	**SD**	**P value**	**Std. Error of the Mean**
Before installing the purifier device	.3150	.03704	0.001	.01512
After installing the purifier device (ppm)	.0497	.07426	0.001	.03032

* p< 0.05 is statistically significant.

Another round of sampling was done 6 months later from the same filters of home water purifier. Details are illustrated in Table 3 and 4.

**Table 3:** The amount of fluoride in tap water after 6 months of using a water purification filter

**Amount of Fluoride** **Water Purification Devices**	**Before using home water purifier (ppm)**	**After 6 months of using the same filter ** **(ppm)**
Alkusar	0.283	0
Aquafresh	0.310	0.089
Soft Water	0.315	0
Water Safe	0.285	0
Puricom	0.312	0
CCK	0.385	0

**Table 4:** Comparison of the study groups after six mounts with t-test

	**Mean**	**SD**	**P value**	**Std. Error of the Mean**
Before installing water purifier	.0497	.07426	0.00	.03032
After 6 months of using the same filter	.0133	.03266	0.00	.01333

## Discussion

Fluoride absorption is mostly systemic or local; systemic absorption occurs through eating the element with food, water or fluoride pills, and local absorption by toothpastes and other fluoride-containing hygienic products. In many countries, the highest supply for fluoride absorption is systemic absorption through water consumption.(6) In early 20^th^ century, the first attempts were made to fluoridate public water supplies, which eventually led to 40% decrease of dental caries in the target population.(7)Introduction of water fluoridation in the 1950-1960 and fluoride-containing dental products in the 1970 changed the situation. The main sources of fluoride in established market economies (EME) are drinking water, fluoridated salt, foods and beverages, baby cereals and formulas, fluoride supplements, toothpastes, mouth-rinses, and topical fluorides. Additionally, fluoride in water has a diffusion or halo effect; which means that the drinks and foods manufactured in fluoridated areas are also available to whole population including the residents of non-fluoridated areas.

Although adding fluoride to almost all oral hygienic products has restricted the effect of fluoride water (Halo effect), it is still common to fluoridate the city water supply.(6) In many areas of the world, there is no systematic plan for fluoridation of community water and only the natural sources supply it. Therefore, sometimes the hardness of water and aggregation of different and sometimes poisonous elements drive the population to use bottled water or use home purification devices.

The findings of the present study revealed that all the 6 devices reduced the fluoride in tap water and most of them nearly eliminated it. Different home purification devices have been marketed each of which is claimed to eliminate certain kinds of elements from water.(9) JK Mwabi *et al.* (2011) used 4 different filters to reduce the hardness and chemical contamination of water in poor villages in Africa, and reported that all of the four filters reduced the fluoride significantly. Bucket filter had the most significant effect and reduced fluoride element 99.9%. These results also indicated that fluoride was the most reduced element of all. Likewise, silver-impregnated porous pot (SIPP) filter reduced 90%-100% of elements.

Clasen *et al.*(5) in their study reported that 3 different home purification systems ,the ceramic candle gravity filter, iodine resin gravity filter, and iodine resin faucet filter, reduced bacterial contamination by four logs and decreased ions such as fluoride and arsenic, as well.

Moreover, there are certain methods to reduce the excessive amount of fluoride in the water. One of the best-known methods is absorption technique.(7) Evaluation of 6 different commercial water purifiers has not been done in any other study; therefore, there is no similar study to compare the results exactly. More evaluations are suggested to be performed on home water purification systems, and more strategies should be devised to preserve the essential elements of tap water.

## Conclusion

The current study found considerable differences between the amount of fluoride before and after filtration with home purification device; that is filtration significantly decreased the fluoride concentration even as much as 100% in some cases. 
